# Implementation of WHO guidelines on management of advanced HIV disease and its impact among TB co-infected patients in Tanzania: a retrospective follow-up study

**DOI:** 10.1186/s12889-022-13498-x

**Published:** 2022-05-27

**Authors:** Frank E. Hassan, Mbazi Senkoro, Nicholaus P. Mnyambwa, Amani Wilfred, Síle F. Molloy, Harrieth Manisha, Sokoine Kivuyo, Sayoki G. Mfinanga

**Affiliations:** 1 National Institute for Medical Research- Muhimbili Research Centre, Dar es Salaam, Tanzania; 2 Institute for Infection and Immunity, St George’s University of London, London, UK

**Keywords:** AHD, TB, HIV, WHO, AHD guideline

## Abstract

**Background:**

The commonest causes of mortality in people living with HIV (PLHIV) are preventable and the majority can be attributed to undiagnosed tuberculosis (TB). National HIV/AIDS control programs are encouraged to implement the WHO package of interventions to improve survival among PLHIV. We assessed the implementation of the WHO TB-related package of care for Advanced HIV Disease (AHD) and its impact on treatment outcomes among HIV/TB patients in Tanzania.

**Methods:**

A retrospective cohort study was employed among HIV/AIDS patients on antiretroviral therapy from 21 public health facilities in three regions (Dar es Salaam, Coastal, and Morogoro) of Tanzania. Patients enrolled in care between January 2013- June 2017 (before the introduction of the WHO guidelines) and July 2017-Sept 2018 (during the implementation of the guidelines) were recruited. Data abstraction was done from patient hospital files using a structured questionnaire uploaded on a tablet.

**Results:**

Data from 2624 patients records were collected. Overall, 50% of patients with HIV had AHD with 7.8% of these co-infected with TB. Among AHD participants, 58.3% were female, 80.7% were from urban areas and 40.0% visited care and treatment centres as self-referrals. Implementation of the WHO AHD package of care was very low, ranging from 0% for Urine LF-LAM test done among patients with symptoms and signs of TB to 39.7% AHD concurrent with TB patients whose ART initiation was deferred for 2 weeks. Overall, the Proportion of AHD patients diagnosed with TB was 4.8%, Of which sputum Xpert as the first test for TB diagnosis was 4.4%. Five patients (0.6%) were documented to have received IPT at enrolment. Tailored counselling to ensure optimal adherence to ART for viral suppression was given to 12.1%.

AHD patients co-infected with TB were retained in care more before the introduction of WHO AHD guideline (82.1%) compared to the period after the introduction of the guideline (53.9%) (*p* = 0.008). Clinical failure at 6 months among AHD patients was 10.6% before the guideline and 11.4% after the guideline. Immunological failure was observed in 1 patient (9.1%) before the guideline and 1 patient (7.1%) after the guideline. After the introduction of the guideline, mortality was 5.9% and no mortality was observed before the guideline. All the differences were not statistically significant.

**Conclusions:**

Implementation of the TB related WHO packages of care for AHD is very low. Except for TB diagnosis, other parameters did not improve with the introduction of the guidelines. More research is recommended to ascertain the effectiveness of guidelines as well as an understanding of the mechanisms involved.

## Background

Despite significant advances in earlier initiation of antiretroviral therapy (ART) and improved access to HIV testing and treatment, over a third of people living with HIV (PLHIV) in low- and middle-income countries (LMICs) present to care with Advanced HIV Disease (AHD) [1]. The World Health Organization (WHO) defines AHD in adults, adolescents, and children older than five years as having a CD4 cell count < 200cells/mm.^3^ or stage 3 or 4, including both ART naïve individuals and those who interrupt treatment and return to care; all children younger than five years with HIV are considered as having AHD [2]. The commonest causes of mortality in PLHIV are preventable [3–5] and the majority can be attributed to undiagnosed tuberculosis (TB) [4], which accounts for about 27% of all AIDS-related deaths globally [4]. In 2015, TB alone accounted for one-third of the estimated 1.1 million AIDS-related death globally, with most of these TB-associated deaths (200 000 cases) occurring among men [9]. Similarly, in 2017, an estimated 300 000 (range, 266 000–335 000) deaths among HIV-positive individuals were associated with TB [10]. In sub-Saharan Africa, TB has been reported as a leading cause of morbidity and mortality among adults and children living with HIV [9] and it was accounted for approximately 84% of all deaths from HIV-associated TB in 2018 [6]

The burden of both HIV and TB is very high in Tanzania, and the country remains among the top 15 countries with a high number of PLHIV in the world [7]. The 2011–2012 Tanzania HIV/AIDS Indicator Survey reported 5.1% of the adult population were infected with HIV, with 36% of TB patients co-infected with HIV [8].

In 2017, WHO published guidelines for managing AHD [9]. A package of interventions to reduce mortality and morbidity was recommended, based on the results of two randomized trials, both showing a mortality reduction associated with the delivery of a simplified intervention package [10]. The package includes (i) screening, treatment, or prophylaxis, or a combination, for major opportunistic infections such as TB, (ii) rapid ART initiation, and (iii) intensified adherence support for everyone presenting with AHD [2]. The guideline describes packages related to TB disease among advanced HIV patients to involve (i) Sputum Xpert MTB/RIF as the first test for TB diagnosis among symptomatic people, (ii) Lateral Flow Urine Lipoarabinomannan Assay (LF-LAM) for TB diagnosis among people with symptoms and signs of TB, (iii) TB preventive treatment (IPT) offered to all patients regardless of CD4, (iv) Defer initiation of ART for symptomatic TB patients, and (v) Tailored counselling to ensure optimal adherence to the advanced disease package [9].

National HIV programs are encouraged to implement the WHO package of interventions to improve survival. Although Tanzania immediately started the implementation of the guidelines in 2017, its implementation and impact remain unassessed. Hence, the present study aimed to evaluate the degree of implementation of the WHO recommended package of care for AHD patients in relation to TB and its impact on outcomes among HIV-infected patients co-infected with TB in Dar es Salaam, Pwani, and Morogoro regions in Tanzania.

## Methodology

### Study design and setting

This was a hospital-based, retrospective cohort study. Data abstraction was implemented from December 2019 to June 2020 for HIV patients enrolled in Care and Treatment Clinics (CTC) from January 1, 2013, to September 2018. HIV patients transferred in or out and those with prior-ARV exposure were excluded from this study. Twenty-one health facilities (Table 1) in Dar es Salaam, Pwani, and Morogoro regions where another project titled TRIP was ongoing were included. TRIP was a translational study of an evidenced-based innovative REMSTART package to reduce mortality in advanced stage HIV patients starting ART in Tanzania [11]. We used the regions where the TRIP project was taking place to ease logistics and implementation of the current study since an enabling environment was already present.

**Table 1:** Selected facilities

Facility	Facility level	Location (Urban/Rural)
Amana Hospital	Regional hospital	Urban
Mnazi mmoja Hospital	Hospital	Urban
Vingunguti Dispensary	Dispensary	Urban
Tabata A Dispensary	Dispensary	Urban
Kitunda Dispensary	Dispensary	Urban
Buguruni Health centre	Health centre	Urban
Mwananyamala Hospital	Regional hospital	Urban
Shree Hindu Mandal	Private Hospital	Urban
TMJ Hospital	Private Hospital	Urban
Hubert Kairuki Memorial Hospital	Private Hospital	Urban
Tandale Health centre	Health centre	Urban
Bunju Dispensary	Dispensary	Urban
Morogoro regional Hospital	Regional hospital	Rural
Sabasaba Health centre	Health centre	Rural
Mafiga Health Centre	Health centre	Rural
Kilosa District Hospital	Hospital	Rural
Kidodi health centre	Health centre	Rural
Mlandizi health centre	Health centre	Rural
Magindu Dispensary	Dispensary	Rural
Mkuranga district hospital	Hospital	Rural
Kisiju Dispensary	Dispensary	Rural

A multistage sampling procedure was used to select study participants. Of the selected regions, Dar es Salaam, Pwani and Morogoro, two districts were selected from each. Selection of health facilities considered inclusion criteria for health facilities i.e., providing HIV care service from at least 2013. The number of patients obtained from each facility was determined by sample proportion to size allocation technique. At the facility level, a list of all patients (from January 1, 2013, to September 2018) was obtained and simple random sampling using a computer system (Ms-Excel) was used to select patients.

### Data collection

Before the beginning of data collection, all team leads and data abstractors were trained on the protocol, CRFs, and study procedures. A pre-tested structured questionnaire was used for data collection.

Data abstraction was done using programmatic and clinical data; existing care and treatment medical records at the facilities including registers, patient cards (CTC cards), and an electronic database (CTC02 database). The data collection tool was through electronic data capture using a web-based questionnaire tool that was linked to the server at NIMR-Muhimbili. Each data collector had an account and used an encrypted password to access the system and fill out the questionnaire. Data were uploaded directly to the server for storage purposes. The database was also encrypted to make sure confidentiality of collected data was maintained. Access to the server was limited only to authorized personnel.

### Data management and analysis

All necessary checks were embedded in the tool developed in the computer system to reduce errors during data entry. For free entry fields, abstractors ensured completeness of data by reviewing the data before uploading it to the server. Data were further verified for consistency prior to analysis. Analyses were performed using STATA version 14.1. Summary statistics were provided depending on the nature of the variable i.e., for categorical variables, proportions and percentages were reported, for continuous variables median and interquartile range were reported. The percentage of missing data is indicated in the table of baseline characteristics and all analyses were completed on a complete case record basis. Chi-squared and Fisher’s exact tests were used to determine associations between WHO-recommended TB services for AHD patients and time periods before and after the introduction of the guideline with *p*-value < 0.05 were considered significant. Multivariable analysis was done to assess the confounding effect of age, sex, and location on implementation of the guideline, but none of the variables showed the effect.

This manuscript describes TB related recommended WHO package of care for AHD patients. These are 1) Sputum Xpert as the first test for TB diagnosis in symptomatic patients 2) Tailored counselling to ensure optimal adherence to advance disease care package, including home visits if feasible [enhanced adherence counselling (EAC)] 3) Use of Urine LF-LAM for TB diagnosis in patients with symptoms and signs of TB 4) Deferring ART initiation in AHD patients with clinical symptoms suggestive of TB 5) Provision of Isoniazid Preventive Therapy at enrolment. Routine TB diagnosis in selected facilities is mainly through sputum Gene Xpert. In this study, we collected TB status as with TB or without TB.

## Definitions

*Clinical Failure* was defined as a new clinical condition indicating severe immunodeficiency (with the exception of TB and WHO clinical stage 4) after 6 months on antiretroviral therapy.

*Immunological Failure* was defined as CD4 count falling to the baseline (or below) or persistent CD4 levels below 100 cells/mm3 after 6 months for two consecutive follow-up times. We also used viral load levels as defined by WHO guidelines [12]

*Mortality* was defined as participant death from all causes at 12 months follow up period.

## Results

**Fig. 1:**
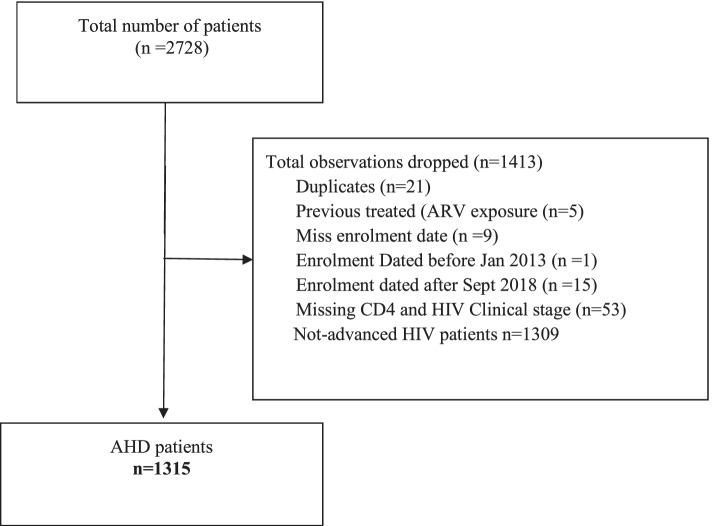
Flow chart for data abstraction

A total of 2728 patient records were abstracted;1413 records were excluded (Fig. 1) including 53 due to missing information on advanced HIV status.

Overall, the median age (IQR) of AHD patients was 37.4 (12.5%) years, similar before and after the introduction of the guideline (Table 1). More patients were female (58.8%) and a majority (80.7%) were from urban areas. Most patients visited care and treatment centres as self-referral (45%). Among participants with AHD, 7.8% were diagnosed with TB during enrolment to HIV care (Table 2).

**Table 2:** Baseline demographic characteristics of AHD patients (2013–2018), *N* = 1315

Characteristic	Before guideline for management of AHD (*n* = 587) n (%)	After introduction of guideline for management of AHD (*n* = 728) n (%)	Total (*N* = 1315) n (%)
**Age (years), *n* = 1293**			
0–14	35 (6.0)	43 (5.9)	78 (5.9)
15–24	22 (3.8)	30 (4.1)	52 (4.0)
25–34	152 (25.9)	165 (22.7)	317 (24.1)
35–44	205 (34.9)	240 (33.0)	445 (33.8)
45–54	110 (18.7)	156 (21.4)	266 (20.2)
55–64	38 (6.5)	68 (9.4)	106 (8.1)
65 +	14 (2.4)	15 (2.1)	29 (2.2)
Missing	11 (1.9)	11 (1.5)	22 (1.7)
Median age (IQR)	38 (31, 46)	40 (32, 48)	39 (31, 47)
**Sex**			
Male	221 (37.7)	317 (43.5)	538 (40.9)
Female	359 (61.2)	408 (56.0)	767 (58.3)
Missing	7 (1.2)	3 (0.4)	10 (0.8)
**Location of district**			
Rural	122 (20.8)	132 (18.1)	254 (19.3)
Urban	465 (79.2)	596 (81.9)	1061 (80.7)
**Referral**			
OPD	130 (22.2)	287 (39.4)	417 (31.7)
MCH	10 (1.7)	15 (2.1)	25 (1.9)
Self-referral	298 (50.8)	228 (31.3)	526 (40.0)
TB/DOTS	49 (8.4)	41 (5.6)	90 (6.8)
other	48 (8.2)	74 (10.2)	122 (9.3)
Missing	52 (8.9)	83 (11.4)	135 (10.3)
**TB Status**			
TB patient	45 (7.7)	57 (7.8)	102 (7.8)
Not TB patient	531 (90.4)	647 (88.9)	1178 (89.6)
Unknown	11 (1.9)	24(3.3)	35 (2.7)

### Implementation of the TB related WHO packages of care for AHD

Proportions of AHD patients receiving TB services before and after the introduction of the WHO package of care for AHD are shown in Table 3. Nearly all AHD patients were screened for TB before and after the introduction of the guideline and the proportion of those diagnosed with TB was almost equal in both periods. The proportion of AHD patients receiving IPT at enrolment was very low (< 1%) in both periods. Differences observed in TB services received by AHD patients between the two periods were not statistically significant even after adjusting for age, sex, and location.

**Table 3:** Implementation of the TB related WHO packages of care for AHD patients before and after the introduction of WHO guideline for the management of Advanced HIV Disease, *N* = 1315

TB service provided	Overall (July 2013-Sept 2018)	Before guideline for management of AHD (Jan 2013- June 2017) n (%)	During guideline for management of AHD (July 2017-sept 2018) n (%)	*P*-value
**TB diagnosis**				
TB patient	102 (4.8)	45 (7.8)	57 (8.1)	0.852
Not TB patient	1178 (88.9)	531 (92.2)	647 (91.9)	
**Total**	**1280**	**576**	**704**	
**IPT at enrolment**				
Received IPT	5 (0.6)	4 (0.9)	1 (0.2)	0.155*
No IPT	893 (67.9)	421 (99.1)	472 (99.8)	
Total	**898**	**587**	1253	
**Sputum Xpert as the first test for TB diagnosis**				
Tested	**57 (4.4)**	**21 (3.7)**	36 (5.0)	**0.146**
Not tested	**1233 (95.6)**	**553 (96.3)**	680 (95.0)	
Total	**1290**	**574**	716	
**Tailored counselling^**				
Counselled	**16 (12.1)**	**7 (10.4)**	9 (13.9)	**0.37**
Not Counselled	**116 (87.9)**	**60 (89.6)**	56 (86.2)	
Urine LF-LAM test				
Tested	**0**	**0 (0)**	0 (0)	
Not Tested	**1275**	**564 (100)**	711 (100)	
ART initiation (TB patients)				
Less than 2 weeks	**85 (60.3)**	**35 (62.5)**	50 (58.8)	** < 0.398**
More than 2 weeks	**56 (39.7)**	**21 (37.5)**	35 (41.2)	
ART initiation (non-TB patients)				
Less than 2 weeks	**932 (86.3)**	**381 (77.0)**	551 (94.2)	** < 0.001**
More than 2 weeks	**148 (13.7)**	**114 (23.0)**	34 (5.8)	

^*^Fisher’s exact test was used to calculate p-value

*^* Tailored counselling is given to HIV patients who fail to attain viral load suppression at a specified time (in this case 6-month from ART start)

Table 3; The results for implementation of the WHO-recommended TB packages of care for AHD patients show during guideline implementation; AHD patients diagnosed with TB (8.1%), 0.2% received IPT, 5.0% Sputum Xpert was the first test for TB diagnosis, 13.9% received tailored Adherence counselling, 0.0% Urine LAM test conducted, 39.7% of AHD patient concurrent with TB were initiated ART more than 2 weeks from enrolment to care.

### Retention of AHD patients coinfected with TB

When we looked at the proportion of advanced HIV patients with TB retained in care for 12-months (*n* = 78), we observed that more AHD patients with TB were retained in care (for at least 12 months) before the introduction of the WHO AHD guideline, 32 patients (82.1%), compared to the period after the introduction of the guideline, 21 patients (53.9%), (*p* = 0.008) (Table 4). When adjusted for sex, the difference between females’ retention was observed to be statistically significant, being 92% before the guideline and 56% after the guideline (*p*-value = 0.04). When adjusted by age, age group 15–55 years were retained more (82.4%) before the introduction of the guideline compared to after (54.6%) (*p* = 0.014). No statistically significant difference was observed when adjusted by location. (Table 5).

**Table 4:** Proportion of AHD patients with TB retained in care (12-Months) before and after the introduction of the WHO package of care for AHD (*N* = 78)

Retention in care	Overall (July 2013-Sept 2018)	(Jan 2013-June 2017)	(July 2017-Sept 2018)	*P*-value
Retained	53 (68.0)	32 (82.1)	21 (53.9)	0.008
Not Retained	25 (32.0)	7 (18.0)	18 (46.2)	
**Total**	**78**	**39**	**39**

**Table 5:** Proportion of AHD patients with TB retained in care (12-Months) before and after the introduction of the WHO package of care for AHD stratified by age, sex and location of district, *n* = 78

Retention in care	Overall (July 2013-Sept 2018)	Before guideline for AHD guideline (Jan 2013-June 2017)	During guideline for AHD guideline (July 2017-Sept 2018)	*P*-value
**Sex**				
**Males, *n* = 47**				
Retained	31 (66.0)	19 (79.2)	12 (52.2)	0.051
Not Retained	16 (34.0)	5 (20.9)	11 (47.8)	
**Females, *n* = 29**				
Retained	21 (72.4)	12 (92.3)	9 (56.3)	0.044*
Not Retained	8 (27.6)	1 (7.7)	7 (43.8)	
**Age **^**b**^				
**15–54, *n* = 67**				
Retained	46 (68.7)	28 (82.4)	18 (54.6)	0.014
Not Retained	21 (31.3)	6 (17.7)	15 (45.5)	
**55 + , *n* = 8**				
Retained	4 (50.0)	3 (75.0)	1 (25.0)	0.486*
Not Retained	4 (50.0)	1 (25.0)	3 (75.0)	
**Location of a district**				
**Rural, *n* = 21**				
Retained	11 (52.4)	7 (77.8)	4 (33.3)	0.08*
Not Retained	10 (47.6)	2 (22.2)	8 (66.7)	
**Urban, *n* = 57**				
Retained	42 (73.7)	25 (83.3)	17 (63.0)	0.131*
Not Retained	15 (26.3)	5 (16.7)	10 (37.0)	

^*b*^* there were 3 patients aged below 15 years, 1 in before introduction of the guideline era and 2 after introduction of the guideline, all of them retained in care*

### ART outcome of AHD patients coinfected with TB

Clinical outcomes of AHD patients who were co-infected with TB are shown in Table 6. There was no difference, in terms of clinical and immunological failures at 6 months, between the periods before and after the introduction of WHO guidelines. There was also no difference between the two periods when we compared all-cause mortality.

**Table 6:** ART outcome of AHD patients with TB, before and after the introduction of the WHO package of care for AHD

Characteristics	Overall (Jan2013-Sept 2018)	Jan 2013- June 2017)	July 2017-sept 2018)	*P*-value
**Clinical failure at 6 months**	**n (%)**	**n (%)**	**n (%)**	
Not failed	81 (89.0)	42 (89.4)	39 (88.6)	0.912
Failed	10 (11.0)	5 (10.6)	5 (11.4)	
Total	91	47	44	
**Immunological failure at 6 months**				
Not failed	23 (92.0)	10 (90.9)	13 (92.9)	1.00 ^a^
Failed	2 (8.0)	1 (9.1)	1 (7.1)	
Total	25	11	14	
**All causes mortality**				
Died	5 (3.6)	0 (0)	5 (5.9)	0.156 ^a^
Alive	134 (96.4)	54 (100)	80 (94.1)	
Total	139	54	85	

^a^ Indicates Fisher exact test was used to measure the association

## Discussion

This study was conducted to look at the implementation of the recommended WHO TB package of care for AHD and its impact on TB/HIV services. Clinical outcomes among HIV-infected patients co-infected with TB was also assessed. Generally, we found the implementation of the TB related WHO packages of care for AHD to be very low. There was a slight increase in TB diagnosis and the use of sputum Xpert as the first test for TB diagnosis during the guideline implementation period. Provision of TB Preventive Therapy at enrolment was low both in the periods before and after the introduction of the WHO Advanced HIV packages. Deferring ART initiation for 2 weeks among TB patients was poorly implemented with a slight increase during the guideline implementation period. We also found no difference in clinical outcomes between the two periods.

The low level of implementation of the TB related WHO packages of care for AHD has also been shown by others. For example, Singhroy et al. showed that, despite positive WHO recommendations and evidence demonstrating that LAM testing for TB reduces mortality in the intended use population, its use remained low among high TB/HIV burden countries [13]. Others have also shown uptake or demand for the AHD package in many low-income and middle-income countries to be low [14]. Some critical enablers of implementing the package of care for AHD have been suggested and can be useful in the Tanzanian context. Such enablers include supporting the routine use of the AHD package both in hospitals and at decentralized primary care clinics and peripheral sites by facilitating task-shifting to nurses and other mid-level healthcare workers [14]

Screening for opportunistic infections, such as TB is crucial because of the high mortality associated with these co-infections in advanced HIV patients. Our findings show that there was no difference in the proportions of patients screened for TB when we compared the periods before and after the introduction of WHO packages of care for AHD in Tanzania. Similar results, of no difference between the periods before and after the introduction of WHO packages of care for AHD, were found for the proportions of AHD patients provided with IPT at enrolment, with the proportions being very small (less than 1%) in both periods. WHO recommends that all PLHIV who are unlikely to have active TB should receive at least 6 months of IPT as part of a comprehensive package of HIV care. The effects of IPT augment the effects of ART on reducing the incidence of TB. The similarity of lack of difference for TB screening and provision of IPT between the two study periods might be due to the fact that procedures for both, TB screening and provision of IPT, remained the same in the two periods. Other studies have shown similar results [15]

Even though the difference in TB diagnosis was not statistically significant, there were more cases of TB diagnosed after the introduction of the WHO packages of care for AHD compared to the period after. This can be a result of a change in screening procedures to include all AHD patients after the introduction of WHO packages of care for AHD. Before the WHO packages of care for AHD, symptomatic screening of PLHIV for TB symptoms was the main alert for initiation of the diagnostic process.

In 2015, WHO issued guidance on the use of the inexpensive urine TB LAM lateral flow assay to assist in the screening and diagnosis of tuberculosis, and this was included in WHO packages of care for AHD. However, evidence from our study and other literature [13] have shown LAM testing for TB remains low, and this might have negatively influenced the number of TB cases identified.

Retention in care of patients with advanced HIV co-infected with TB is another factor that was different between the two study periods, i.e., before and after the introduction of WHO packages of care for AHD. Surprisingly, retention was higher before the introduction of the WHO packages of care for AHD indicating that the introduction of tailored counselling as part of WHO packages of care for AHD, which aims to ensure optimal adherence to the advanced disease package, was ineffective. But other studies have shown that low retention rates in ART care are a result of complex and multidimensional factors such as inadequate space in the clinic, long waiting times, long travel distances, food shortages and patients.

The treatment outcome of TB/HIV co-infected patients is still unsatisfactory as has been shown in other studies [16, 17]. Our results showed that there was no difference in clinical outcomes of advanced HIV patients who were co-infected with TB when we compared the period before and after the introduction of WHO packages of care for AHD. However, interpretation of these results should be done with caution as this could be a result of small sample size and the consequence of missing information in the records reviewed.

The strength of this study was that it included facilities from both rural and urban settings. The study's main limitation involves a high level of missing data from source documents. The effect of missing data could be mitigated during the analysis.

## Conclusion

Implementation of the TB related WHO packages of care for AHD was very low. With the exception of TB diagnosis, other parameters did not improve with the introduction of the guidelines. More research is recommended to ascertain effectiveness of guidelines as well as an understanding of the mechanisms involved.

## Data Availability

The datasets used and/or analysed during the current study are available from the corresponding author on reasonable request.

## Abbreviations

AHD: Advanced HIV Disease
TB: Tuberculosis
HIV: Human Immunodeficiency Virus
LF-LAM: Lateral Flow Lipoarabinomannan
PTB: Pulmonary Tuberculosis
DNA: Deoxyribonucleic acid
